# Dr. Woodforde, on the Influenza

**Published:** 1803-06-01

**Authors:** James Woodforde

**Affiliations:** Ansford


### *To the Editors of the Medical and Physical Journal.*

Gentlemen,

AGREEABLY to the request mentioned in your Journal, I have presented you with a concise account of the Epidemic Catarrhal Fever, as it has appeared in an extensive eastern part of the county of Somerset. The appearance of the Influenza in England was very sudden, and its attack extremely general, so that it is difficult to say, in what, or in how many parts of the kingdom it prevailed at first. It must naturally attract attention, first in the metropolis, and other cities, from their corresponding population and greater number of the sick; this circumstance seems to have given rise to a precipitate conclusion that these were the places first attacked, and that from these it was diffused progressively throughout all others. Whereas, it is more probable that the disease broke out in all the places, at or nearly the same time. The literary history of the Influenza, given by different writers, shews it to be more sudden in its attack, more rapid in its progress, and more universally diffused, than any other epidemic with which we are acquainted, whether contagious or not. Dr. Glass says, when it prevailed at Exeter in the year 1729, it was conjectured that two thousand persons at least were seized with it in one night; and that in the year 1557, according to Mercatus, it attacked all parts of Spain at once, so that the greatest part of the people in that kingdom were seized with it almost on the same day. It is thought by some that the Influenza spreads more generally in towns and cities, than in villages; but I think otherwise, if proper allowance were made for the greater population in the former than in the latter. There has always been a necessary distinction between epidemic diseases that are not contagious, and those that are both epidemic and contagious. Of the first kind we consider Intermittent Fevers, which may be very general, but not contagious; and from the considerations hereafter mentioned, I am inclined to believe the same of the Influenza.

This disease, so frequent in its recurrence, so general in its seizure, and often so violent and dangerous in its consequences, naturally engages the attention of the public, as wells as physicians, and prompts them to investigate its nature and causes. The question agitated for determination, is, whether the Influenza is, or is not contagious? A

question certainly of great consequence, since if it were proved in the affirmative, it might lead to the discovery of prophylactic or preventive means. When I consider the sudden manner in which the Influenza makes its appearance, the great and wonderful rapidity with which it spreads; that it attacks whole counties, nay even whole kingdoms, almost in the same day; that it affords no certain traces of progressive communication from one person to another; and that, in these circumstances, it is peculiar to itself, and different in all respects from other infectious diseases, I am of opinion that it is not contagious; at least, not primarily so; but that it owes its source to the state of the atmosphere. Dr. Darwin observes, "that this malady attacks so many at the same time, and spreads gradually over so great an extent of country, that there can be no doubt but that it is disseminated by the atmosphere. In the year 1782, the sun was for many weeks obscured by a dry fog, and appeared red as through a common mist; the material which thus rendered the air muddy, probably caused the Epidemic Catarrh which prevailed in that year, and which began far in the north, and *extended itself over all Europe.*" Zoonomia, c. ii. 1. 3.

We come now to a more satisfactory subject, a description of the phenomena of the disease in its commencement, progress, and termination, with the medical treatment. The first case of Influenza to which I was called, occurred about three weeks since; from which time the disease has continued to spread, and prevails generally even at this period. Though there are some leading characteristic symptoms in the Influenza, there are others extremely different in different patients, and in all gradations from mildness to severity. A severe case of simple Catarrh presents an imperfect model of a mild one of the Influenza; chilliness, with rigors, sometimes pretty severe, with head-ach, pain in the loins, back and limbs, vertigo, nausea and sometimes vomiting, great and sudden languor, with anxiety, depression of spirits, and debility.—The cold stage is various in duration, and sometimes it does not happen at all, or in so slight a manner as to escape the observation of the sick; the other attendants of fever soon follow, denoted by heat and dryness of the skin, some but little thirst, a brownish fur in the middle of the tongue, but moist at the edges; an early and total loss of distinction of taste, with inappetency, sneezing, a thin acrid defluxion from the nose, with a stuffing in the same, impeding the admission of air; a watery discharge from from the eyes, which are often much inflamed, preternaturally irritable, and extremely painful on exposure to light; a frequent tickling cough, at first dry, and particularly violent on first lying down; it is often as violent and convulsive as in hooping cough, and frequently occasions vomiting and epistaxis. This cough, from being at first dry, becomes gradually moist, and followed by an expectoration, more or less copious, of viscid phlegm and mucous, which is of a whitish or yellowish colour. When the expectoration is copious, and brought up freely, the patient may be considered out of danger, except he be under the existence of phthisis, or any other violent and dangerous disease. The pulse is generally about 80, and I have not known it exceed 100; it is commonly soft and full at the beginning, but in the course of a few days becomes weak. A severe inflammatory affection of the chest sometimes happens, marked by the violence of the cough, dyspnœa, oppression, and pyrexia, and by transient stitches in various parts, and sometimes by a fixed pain in the side. In several patients, of robust and plethoric habits, I have seen all the diagnostic symptoms of pneumonia present, except a direct pain in the thorax.

A sudden prostration of strength, anxiety and despondency take place I believe, in some degree, in every instance of Influenza; and those symptoms mark and characterize it from all other diseases. In several slight cases no other symptoms than those attending a mild catarrh were present, except great debility and lassitude; while, in some instances, the phlogistic diathesis and sanguine congestion in the lungs have passed on to pneumonia; in others, indirect debility has quickly followed, and the disease has assumed the appearance of, and actually terminated in, typhus or low fever, of an uncertain duration and event. When the Influenza was epidemic in 1782, many people labouring under it, often dropped down suddenly on going to work. Many cases have occurred with, and many without, any inflammation of the internal fauces; but most patients have complained of a dryness in the mouth and throat, and soreness in the wind-pipe and œsophagus. With us, the duration of the disease has been from three or four days, in slight cases, to a fortnight or three weeks in severe ones. After the febrile symptoms disappeared, the cough has frequently continued with great severity, and a copious mucous expectoration for some time after, accompanied also with want of appetite, and great languor and debility. I have hardly seen a case but with more or less of cough. When the febrile symptoms have been considerable, the urine has been high coloured, with a lateritious sediment; but in slight cases it has undergone very little change. A diarrhœa, in many cases, either happened spontaneously in the first days, or was easily excited by gentle opening medicines; the nights are generally restless, with little sleep.

As to the treatment of this disease, I have found slight cases, resembling simple catarrh, yield speedily to confinement to the house, lying some hours longer in bed, and the liberal use of diluting, cooling liquids, mild diaphoretics, and gentle and repeated purgatives. In severe attacks, and especially in athletic young men used to full diet and robust exercise, and with symptoms of inflammation or congestion in the head or lungs, a different treatment becomes necessary. Here, a total confinement to bed, with the exception only of sitting up for a few hours at a time, an early and moderate venæsection, a blister to the chest, saline opening medicines, or an emetic, followed by demulcents and gentle diaphoretics, have commonly produced the wished-for effect. The most material points to be determined in the cure of Influenza, are the propriety of venæsection and the use of antimonials. In all acute diseases, and particularly in this, so various, fluctuating, and uncertain in its symptoms, progress, and termination, it is difficult to ascertain the decided efficacy of any remedy, more especially from the practice of any individual: It is by uniting our own observations and experience to that of many others, and by a judicious and careful comparison of the whole, that we can be enabled to lay down any general rules for practice in any disease; and even after all our labours and united exertions, numerous exceptions will present themselves.

I shall now speak of what has and what has not succeeded with me. In all cases where there is not a combination of acute pain in the chest, great dyspnœa, a dry, frequent, and violent cough, a full if not a hard pulse, thirst, considerable heat of skin, and high coloured urine, in fact, the presence of direct symptoms of peripneumony, I conceive the use of the lancet either superfluous, inadmissible, or certainly injurious; and I have seen cases that have demonstrated the one or the other. On the other hand, under the existence of the above symptoms, I have witnessed great, speedy, and decisive benefit from bleeding, repeated even twice or thrice. The state of the blood too, has confirmed the propriety of the operation; for, in every every instance, it exhibited a firm buffy coat, with a cup-like appearance. Antimonials, even in small doses, have not generally succeeded; they have operated too powerfully on the stomach and bowels, in occasioning vomiting or diarrhœa, and frequently both. In some cases, perhaps, this effect arose from the omission of an emetic in the beginning, which I think is often beneficial, and puts the disease in a more favourable state. The saline mixture made with ammonia preparata, or the aq. ammon. acetat. have for the most part answered as diaphoretics. After the first days of the disease, and when the febrile symptoms are abated, the occasional use of anodynes has afforded great relief to the cough and restlessness, without increasing the heat of skin or dyspnœa, or accelerating the pulse. At the same period, in case of great debility, languor, and loss of appetite, much benefit has been derived from gentle not heating tonics; such as, inf. gentian. comp. vel quass. c. vel absque acid. vit. dilut. Though an abstinence from solid meat has been both desired and recommended, yet light preparations from it, in the way of broths and jellies, have been grateful and refreshing, and by a gentle, uniform stimulus, have supported the strength and spirits of the patient under an oppressive disease. I have constantly found wine inadmissible before the subduction of the inflammatory symptoms.

*Ansford,*
*April* 18, 1803.

I am, &c.
JAMES WOODFORDE, M.D.

